# Morphological characteristics and genetic evidence reveals a new species of *Manihot* (Euphorbiaceae, Crotonoideae) from Goiás, Brazil

**DOI:** 10.3897/phytokeys.77.11738

**Published:** 2017-03-17

**Authors:** Marcos José da Silva, Thannya Nascimento Soares, Patrícia Rasteiro Ordiale Oliveira

**Affiliations:** 1 Laboratório de Morfologia e Taxonomia Vegetal, Departamento de Botânica, Universidade Federal de Goiás, CP 131, 74001-970, Goiânia, GO, Brazil; 2 Laboratório de Genética e Biodiversidade, Instituto de Ciências Biológicas, Universidade Federal de Goiás, CP 131, 74001-970, Goiânia, GO, Brazil

**Keywords:** Endemism, Manihoteae, mountainous areas, speciation, taxonomy

## Abstract

During botanical expeditions between 2010 and 2015, as part of a taxonomic study of *Manihot* in the Midwest region of Brazil, approximately 500 specimens of the genus were collected. Some of these specimens presented similarities to *Manihot irwinii*. However, after careful morphological analyses, associated with genetic evidence, we propose here *Manihot pulchrifolius* as a new species. The new species is described, illustrated, and compared to *Manihot irwinii*, its most similar species. Furthermore, geographic distribution, conservation status, and period of flowering and fruiting of the novel species are also provided.

## Introduction

*Manihot* Mill. encompasses over 100 Neotropical species, and therefore stands out as one of the largest genera of Euphorbiaceae in Brazil, with ca. 80 species (Silva 2014). In the Cerrado Biome, over 50 species of *Manihot* have already been documented, among which 40 are endemic (Silva et al. 2013). Nevertheless, recent studies of the genus in the Chapada dos Veadeiros region, in the state of Goiás, Brazil, revealed some new species (Silva et al. 2013, Silva 2014, Silva and Sodré 2014, Silva 2015a, b, Silva et al. 2016a, b), which demonstrates that there is still a lot to discover about this genus, whose taxonomy remains relatively poorly known in the Cerrado Biome. Some species of *Manihot* endemic to the Cerrado Biome have leaves that are considerably diverse morphologically, a fact that has aroused the interest of botanists and geneticists (Duputié et al. 2011, Silva et al. 2016b)

During botanical expeditions to the Serra Dourada State Park, in the state of Goiás, Brazil, since October 2010, as part of a floristic survey of Euphorbiaceae, approximately 500 specimens of *Manihot* were collected, some of them showing similarities to *Manihot irwinii* D.J. Rogers & Appan regarding habit and foliage type. After careful morphological analyses of these collections, associated with genetic evidence, we propose herein the new species *Manihot pulchrifolius*. A detailed description, comments on flowering, fruiting, distribution, environmental preferences, conservation status, and comparisons with morphologically similar species are provided.

## Materials and methods

### *Morphological Studies*

The description of the new taxa was based on observations of populations in the field since 2010, analyses of available specimens from herbaria UFG, NY, K, RB, and UB (acronyms follow Thiers continuously updated), and review of the literature (Rogers and Appan 1973, Allem 1989). The terminology used to describe the types of inflorescences and leaves follows Rogers and Appan (1973). The illustrations were based on fresh material fixed in alcohol 70% during collection in the field. Holotypes of the new species are deposited at UFG, and isotypes are going to be sent to NY, K, RB, and UB. Photographs of natural populations were taken in the field. The conservation status of the species follows IUCN (2016).

### Taxon sampling, DNA extraction, PCR, and sequencing for genetic studies

Leaves were sampled from 126 plants known as *Manihot irwinii* D.J. Rogers & Appan, collected in four localities in the state of Goiás (Table 1). DNA was extracted from silica-dried leaf tissues (Doyle and Doyle 1987) and amplified microsatellite markers by polymerase chain reaction (PCR) using primers GA-12, GA-16, GA-21, GA-126, GA-131, GA-134, and GA-136 developed for *Manihot esculenta* Crantz by Chavarriaga-Aguirre et al. (1998). The amplification was done in 10 μL reactions containing 3 μL of template DNA (1.5 ng/μL), 3 μL of each primer (0.9 μM), 1 μL 10X enzyme buffer containing 2.5 mM MgCl_2_ (500 mM KCl, 100 mg/mL Tris-HCl, pH 8.4, 1% Triton X-100), 1.3 μL bovine serum albumin (10 mg/mL), 0.9 μL 2.5 μM dNTP, and 5 units/μL Taq DNA polymerase, completing the volume with 0.65 μL ultrapure deionized water. PCR parameters were: 94°C for 5 min, 30 cycles of 94°C for 1 min, 56°C for 1min, 72°C for 1 min, and 72°C for 45 s. The forward primers were fluorescently labeled for the observation of fragments of DNA using the Applied Biosystems™ 3500 genetic analyzer (Thermo Fisher Scientific Inc., Waltham, MA, USA) in two multiplex genotyping systems. The genotypes were determined using GeneMapper® Software Version 5.0 (Applied Biosystems™, Thermo Fisher Scientific Inc., Waltham, MA, USA) with default settings.

**Table 1.** *Manihot irwinii* sensu lato collected per site and their geographic coordinates.

Municipalities/Localities	Number of specimens	Voucher/Herbarium	Geographic coordinates/elevation
Mossâmedes/Serra Dourada State Park	29	M.J. Silva 5801/UFG	16°05'35.4"S, 50°11'4.5"W, 972 m
Corumbá de Goiás/100 m above the waterfall Salto de Corumbá	33	M.J. Silva 5805/UFG	15°50'25"S, 48°46'7"W, 1,067 m
Pirenópolis/Serra dos Pireneus, near Serra dos Pireneus State Park	37	M.J. Silva 6389/UFG	15°43'15"S, 49°2'45"W, 730 m
Cocalzinho de Goiás/after Serra dos Pireneus State Park	27	M.J. Silva 6406/UFG	15°46'55"S, 48°50'00"W, 1,157 m
Total	126		

### Data analysis

The genetic diversity of the populations studied was assessed based on estimates of the average number of alleles per locus (A), rarefied allelic richness (AR), observed heterozygosity (Ho), expected heterozygosity under Hardy-Weinberg equilibrium (He), and intrapopulation fixation index (f). The genetic structure of the populations was evaluated according to Weir and Cockerham (1984). These analyses were conducted using the package Hierfstat for the statistical software R (Goudet 2005). The genetic structure was assessed by a Bayesian approach, conducted using the software STRUCTURE 2.3.4 (Pritchard et al. 2000), assuming a model that allows mixing alleles between populations for four independent runs, with K values ranging from one to ten. The tests were performed using the Markov Chain Monte Carlo (MCMC) method, with periods of burn-in of 10,000 and 1,000,000 replicas. The average of likelihood values for each K for all runs was determined by the statistical ΔK developed by Evanno et al. (2005).

The pattern of differentiation among populations was evaluated by calculating the genetic distance between pairs of populations, based on estimates of pairwise by fixation index (F_ST_). To visualize the pattern of differentiation among populations, the genetic distance matrix was subjected to a cluster analysis using the unweighted pair-group method with arithmetic averages (UPGMA). To assess the degree of representativeness of the dendrogram, the cophenetic correlation coefficient was estimated with 10,000 permutations. These analyses were conducted using R Hierfstat (Goudet 2005) and *adegenet* (Jombart 2008).

## Taxonomic treatment

### Manihot pulchrifolius

Taxon classificationPlantaeMalpighialesEuphorbiaceae

M.J.Silva sp. nov.

urn:lsid:ipni.org:names:77161449-1

Figures 1 , 2

#### Type.

BRAZIL. Goiás: Mossâmedes, Serra Dourada State Park, near Pedra Goiana, Cerrado rupestre, on rocky crevices, 16°04'40,5"S, 50°11'20.8"W, 988 m, 21/XI/2014, fl., *M. J. Silva & A. A. Alonso 6232* (holotype: UFG; isotypes: NY, F, K, UB).

#### Diagnosis.

Shrubs up to 2.5 m tall, erect, glabrous; young branches and young leaves reddish to purplish, green-vinaceous to violet; adult leaves 5-lobed at the plant base, 3-lobed along the stem, or rarely unlobed near inflorescence; long racemes or panicles (up to 27 cm long), erect to pendent, axes reddish to purplish; calyx of staminate flowers reddish or purplish with yellow margins, filaments pubescent; bracts and bracteoles of flowers of both sexes reddish to purplish; fruits dark green with violet to purplish wings.

#### Description.

Shrubs 1–2.5 m tall, monoecious, erect; stems and adult branches robust, cinereous, glabrous, waxy, and glossy; young branches and young leaves reddish to purplish, green-vinaceous to violet, including axes of inflorescences and petioles; branches dichotomously branched near apex, sometimes pendent in specimens taller than 1.2 m; latex yellowish, copious; petiole 4.5–13 cm long, robust, canaliculated above, dark purplish to violet. Stipules 1.9–2 cm long, linear, entire to discreetly serrate, caduceus. Leaves in alternate spiral arrangement; lamina firmly chartaceous, glabrous on both surfaces, the basal ones 5-lobed, the others 3-lobed, or unlobed near inflorescence, lobes conspicuously overlapping at sinus, 8.4–15 × 4.5–10 cm, widely oblong-obovate, elliptic-obovate to obovate, apex cuspidate, sometimes oblique, base cordate; venation camptodromous-brochydodromous, primary and secondary veins pinkish, purplish, violet, or rarely yellowish, the primary ones prominent on both surfaces, the secondary ones subparallel to the midrib, impressed on both surfaces, bifurcate or not at apex; adaxial surface dark green and opaque in adult leaves, or light green, castaneous, reddish to purplish in young leaves; abaxial surface opaque green to cinereous with a smooth wax pattern; racemes 5–17 cm long, or panicles 17–24 cm long, laxy, terminal or in the dichotomy of the branches, solitary or in clusters of 2 or 3, erect to pendent, bisexual; if racemes, they have two opposite pistillate flowers at the base and another staminate; if panicles, the secondary axes are similar to racemes, but sometimes without pistillate flowers at the base of secondary axes. Staminate flowers 21–25 mm long; buds 9–10 mm long, globoid to orbicular, reddish, obtuse at apex, glabrous and waxy externally; bracts 11–12 × 2.5–3.3 mm, oval, elliptic, entire, acuminate at apex, reddish, persistent, glabrous on both surfaces; bracteoles 5.7–7 × 1–2 mm, linear, subalternate, distributed on the lower third of the pedicel, glabrous on both surfaces, margins entire, not ciliate, caduceus; pedicels 8.5–12 mm long, cylindrical, reddish to purplish, waxy, glabrous; calyx 12–15 × 10–11 mm, widely campanulate, reddish with yellowish margins, glabrous externally and shortly tomentose internally, lobes widely triangular to ovate, apex obtuse; stamens 10, in two whorls of five, filaments 8–8.2 mm long, pubescent distally, anthers 3–3.2 mm long, oblongoid, yellowish; disk 10-lobed, dark yellow, lobes rounded. Pistillate flowers 19–25 mm long; buds 6–7 mm long, ovoid, glabrous, yellowish with reddish spots; bracts 8–15 × 2.4–3 mm, oval-lanceolate to lanceolate, vinaceous to purplish, entire, acuminate at apex, persistent, glabrous, not ciliate; bracteoles 10–12 × 2–3.5 mm, lanceolate, margin discreetly serrate, similar to the bracts in color; pedicels 13–16 mm long, cylindric-clavate, glabrous, greenish, sepals 8.2–11 × 3.3–5 mm, ovate to oval-elliptic, rarely lanceolate or triangular, shortly pubescent in the upper third internally, apex obtuse, yellowish with reddish pigmentation, margins involute; ovary 3–3.2 × 2–2.1 mm, oblongoid to globoid, glabrous, not winged, green, disk discreetly lobed, yellowish, styles 3, conspicuously united at the base, free portion 2.1–2.2 mm long, with papilose apex. Capsules 12–14 × 16–17 mm, globoid, smooth, glossy, winged, with septicidal and loculicidal dehiscence, green to purplish, slightly glossy. Seeds 8–10 × 4–5 mm, oblongoid, cinereous, with dark spots; caruncle 0.9–1 mm long, sessile, reniform, cream to whitish.

**Figure 1.**
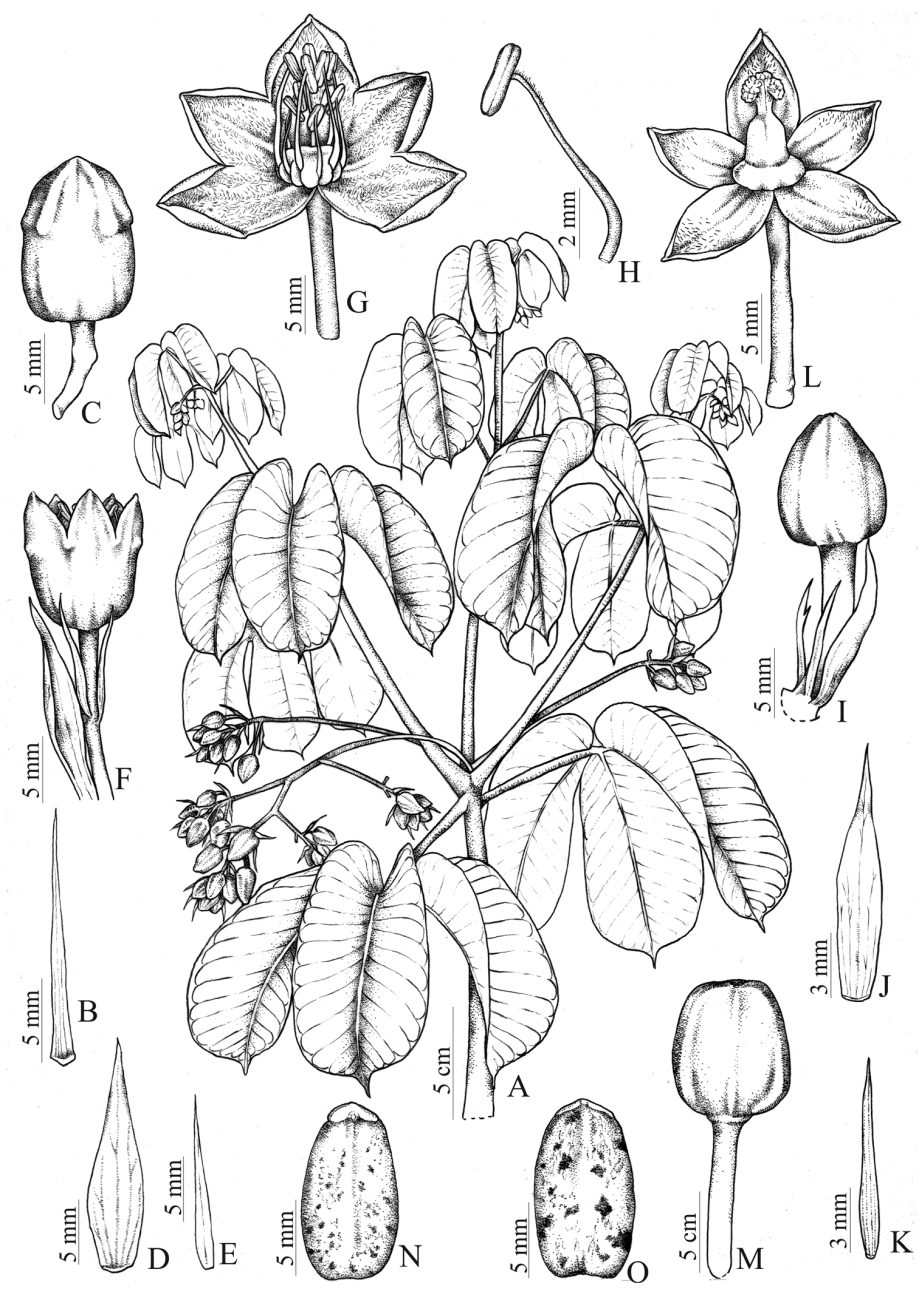
*Manihot pulchrifolius*. **A** Flowering branch **B** Stipule **C** Staminate bud **D** Staminate bract **E** Staminate bracteole **F** Staminate flower **G** Staminate flower with calyx split and open **H** Stamen **I** Pistillate bud **J** Pistillate bract **K** Pistillate bracteole **L** Pistillate flower **M** Fruit **N** Seed, ventral side **O** Seed, dorsal side. Drawn by Cristiano Gualberto from the holotype.

**Figure 2.**
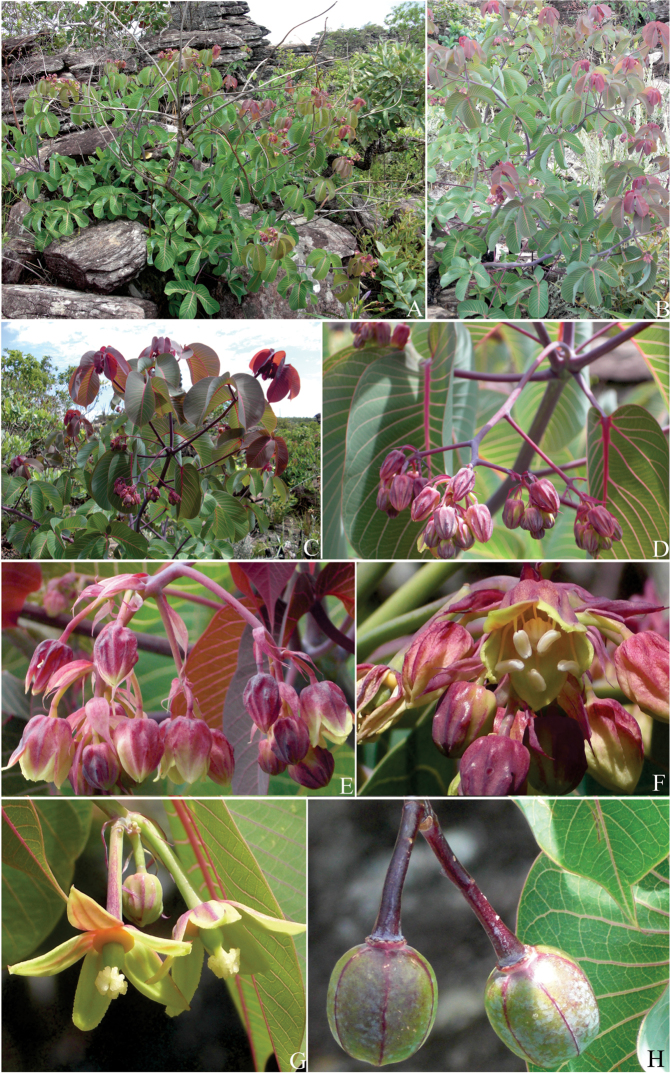
*Manihot pulchrifolius*. **A** Habit; note the plant growing between rocky crevices **B** Habit; detail of the waxy stem **C** Portion of the stem showing inflorescences in clusters at the dichotomy of the branches **D** Adult panicle **E** Portion of the panicle showing the staminate buds and flowers with vinaceous calyx and yellow margins **F** Staminate flowers **G** Pistillate flowers **H** Mature fruits; note the violet wings.

#### Distribution and Ecology.

**Figure 3.**
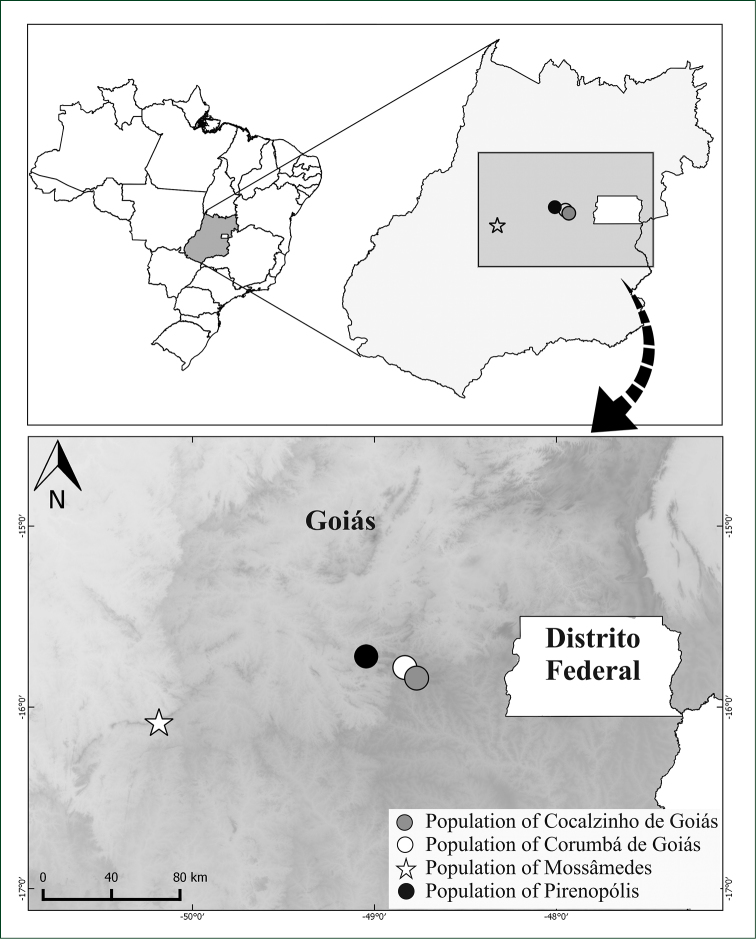
Distribution map of sampled populations highlighting the population of Mossâmedes (= *Manihot pulchrifolius*). The other populations correspond to *Manihot irwinii*.

*Manihot pulchrifolius* is endemic to the state of Goiás, where it was found growing in Serra Dourada (Figure 3), one of the most beautiful and preserved mountainous areas in the state. This mountain range encompasses the Serra Dourada State Park, an area of over 30,000 hectares protected by law since 1965. The species grows in Cerrado *sensu stricto*, on rocky outcrops, rocky slopes, and Cerrado rupestre, in clayey, clayey-stony, and sandy soils, or even on rocky crevices, between 900 m and 1,000 m.

#### Phenology.

The species has been collected with flowers and fruits from November to July. However, the flowers are more usual from January to March, whereas the fruits are more abundant from April to July.

#### Etymology.

The specific epithet “*pulchrifolius*” alludes to the beautiful foliage of the species, especially in the leaf flushing stage, when the leaves are reddish or purplish to green-vinaceous to violet.

#### Conservation status.

Given that the populations have more than 50 individuals and the vegetation where they grow is commonly found in the central part of the state of Goiás (in the municipality of Goiás and neighboring municipalities), we consider *Manihot pulchrifolius* as Least Concern (LC) according to IUCN (2016).

#### Specimens examined.

**BRAZIL. Goiás**: Mossâmedes, Serra Dourada State Park, on the way to Pedra Goiana, 9 Dec 2009, fl., *A. M. Teles 658* (UFG); *ib.*, 30 Oct 2010, fl., *M. J. Silva et al. 3138* and *3139* (UFG); *ib.*, near Pedra Goiana, 16°04'34,7"S, 50°11'28"W, 994 m, 21 Nov 2014, fl., *M. J. Silva & A. A. Alonso 6234* and *6235* (UFG); *ib.*, 16°04'40,5"S, 50°11'20.8"W, 988 m, 21 Nov 2014, fl., *M. J. Silva & A. A. Alonso 6230* and *6231* (UFG); District of Mirandópolis, Rildo Nogueira farm, 27 Nov 2010, fl., *M. J. Silva 3194* and *3198* (UFG); *ib.*, near the first gate that leads to the park headquarters, 16°04'46,9"S, 50°11'28,4"W, 985 m, 29 Jan 2011, fl., fr., *M. J. Silva 3372* (UFG); *ib.*, 4 km above the Piçarrão stream, 16°04'17,4"S, 50°11'26,1"W, 993 m, 30 Apr 2011, fl., fr., *J. E. C. Júnior 15* and *18* (UFG); region after Areal, 16°04'00,9"S, 50°10'8,9"W, 953 m, 28 May 2011, fr., *J. E. C. Júnior 51* (UFG).

## Discussion

### Remarks

*Manihot pulchrifolius* was identified by Rogers and Appan (1973) as *Manihot irwinii* according to the collections *Irwin et al. 12959* (B, F, G, GH, MO, NY, SP, UB, US, and W), *Irwin et al. 11752* (B, F, G, GH, MO, NY, SP, UB, US, and W), and *Macedo 3476* (NY, S, and US). In a taxonomic survey of *Manihot* in the Serra Dourada State Park, Carmo Júnior et al. (2013) adopted the same concept of Rogers and Appan (1973), probably because they were not aware that *Manihot irwinii* is a little known species, scarcely represented in Brazilian herbaria (UB, CEN, HPB, and UFG), and with distribution restricted to the Serra dos Pireneus and neighboring areas where it grows in open areas of Cerrado *sensu stricto* in clayey soils. However, in the last two years, morphological and genetic studies developed by the authors of this paper have shown that populations from Serra dos Pireneus and the Serra Dourada State Park previously identified as *Manihot irwinii* present differences regarding leaf, inflorescence, and flower morphology, as well as genetic structure as described below. Therefore, we concluded that the specimens from the Serra Dourada State Park belong to a new species, herein named *Manihot pulchrifolius*. Both species share a shrubby habit, leaves with lobes overlapping basally, midrib veins thickened, secondary veins subparallel, flowers of both sexes with sepals pubescent internally, and winged fruits.

*Manihot pulchrifolius* has leaves that are 5-lobed at the base of the stem, 3-lobed above the base of the plant or along the plant, or rarely unlobed near the inflorescence, with robust petiole, reddish to dark violet, and leaf blade cinereous, green-opaque, or reddish on lower surface (vs. 3-lobed leaves, and commonly unlobed near inflorescence, thin petiole, greenish, and leaf blade whitish in *Manihot irwinii*), racemes and panicles 5–17 cm and 17–24 cm long, respectively (vs. racemes 7–12 cm long), floral buds reddish (vs. yellowish green to greenish), staminate flowers 21–25 mm long, calyx widely campanulate, filaments pubescent distally, staminate bracts and bracteoles reddish, 11–12 × 2.5–3.3 and 5.7–7 × 0.1–0.2 mm, respectively (vs. staminate flowers 14–15 mm long, calyx narrowly campanulate, filaments glabrous, staminate bracts and bracteoles yellowish green with discreet purplish spots, 11–12 × 2.5–3.3 and 5.7–7 × 0.1–0.2 mm, respectively).

Systematically, the new species can be situated in Manihot section Quinquelobae Pax according to Silva (2014), by having a shrubby habit, leaves widely spaced along the branches, basal petiole attachment, deeply lobed leaf blade, lobes of various shapes (but not linear), monoecious racemose or panicled inflorescences, foliaceous or setaceous bracts and bracteoles, winged or wingless fruits. However, since *Manihot quinquelobae* belongs to a polyphyletic group (Silva et al. unpublished), we prefer not to ascribe *Manihot pulchrifolius* to any sections of the genus.

### Genetic studies

A total of 50 alleles were found for the seven loci evaluated in populations of *Manihot irwinii* sensu lato, ranging from three to eleven alleles per locus. The populations studied showed high and similar genetic diversity, which was also observed in other species of the genus (Roa et al. 2000). Only the population of the municipality of Corumbá de Goiás presented genetic diversity values significantly smaller than the others, suggesting that the population may be suffering from a fragmentation of its habitat since it grows in an area surrounded by agriculture and disturbed by anthropic actions related to tourism. The inbreeding values estimated in the four populations were not significant, indicating adherence to the frequencies expected by Hardy-Weinberg equilibrium for the evaluated loci (Table 2).

**Figure 4.**
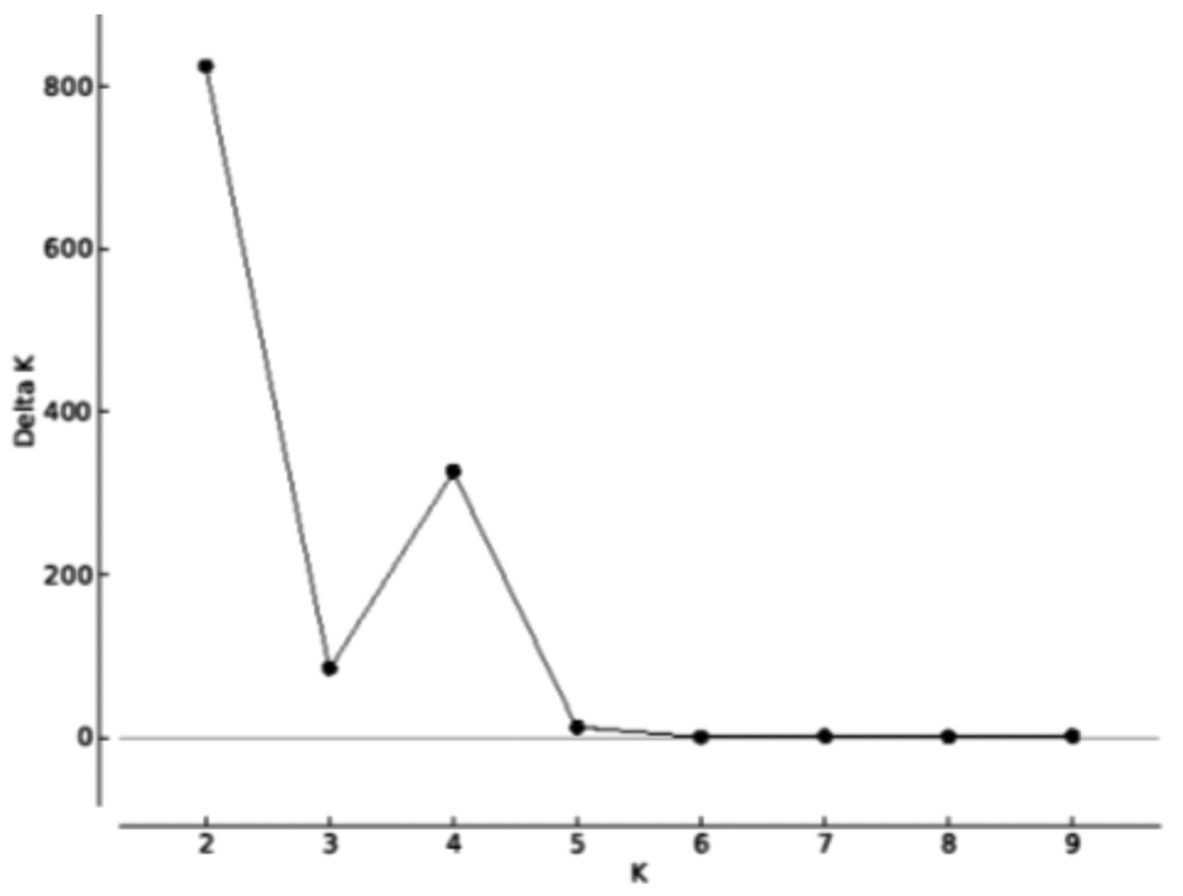
Genetic groups (K) estimated by attribution analysis using the software STRUCTURE. The highest ΔK indicates the most probable number (K) of groups formed by individuals sampled from putative populations of *Manihot irwinii* sensu lato herein analyzed using seven microsatellite markers.

**Figure 5.**
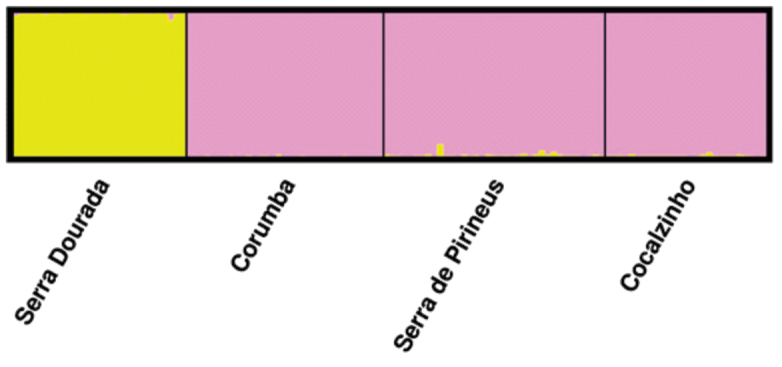
Genetic assignment of four populations of *Manihot irwinii* sensu lato evaluated with seven microsatellite markers, based on Bayesian statistics using the software STRUCTURE.

The population genetic structure analysis showed an estimated value of θ of 0.363, indicating that 36.3% of the genetic diversity is in the component between populations. This level of genetic structure is considered very strong (Wright 1978), especially taking into account the geographical distance among the assessed populations (Figure 3) and the fact that *Manihot irwinii* sensu lato is probably allogamic (Loveless and Hamrick 1984). The estimated values of F (0.359) and f (-0.006, not significant) show that the observed genetic structure is related to the effect of genetic drift and low gene flow between populations and not to the reproductive system of the plant. A strong pattern of genetic structure was also observed in the analysis using the Bayesian approach, which was determined in the formation of two genetic groups (K = 2, Figure 4). The first group determined by STRUCTURE is restricted to the population found in Serra Dourada, municipality of Mossâmedes, whereas the second group contains the other three populations studied (Figure 5). The pattern of attribution of the individuals to the groups was very similar to the patterns observed for different species of the genus *Quercus* that present lack of gene flow between groups (Valencia-Cuevas et al. 2015).

**Figure 6.**
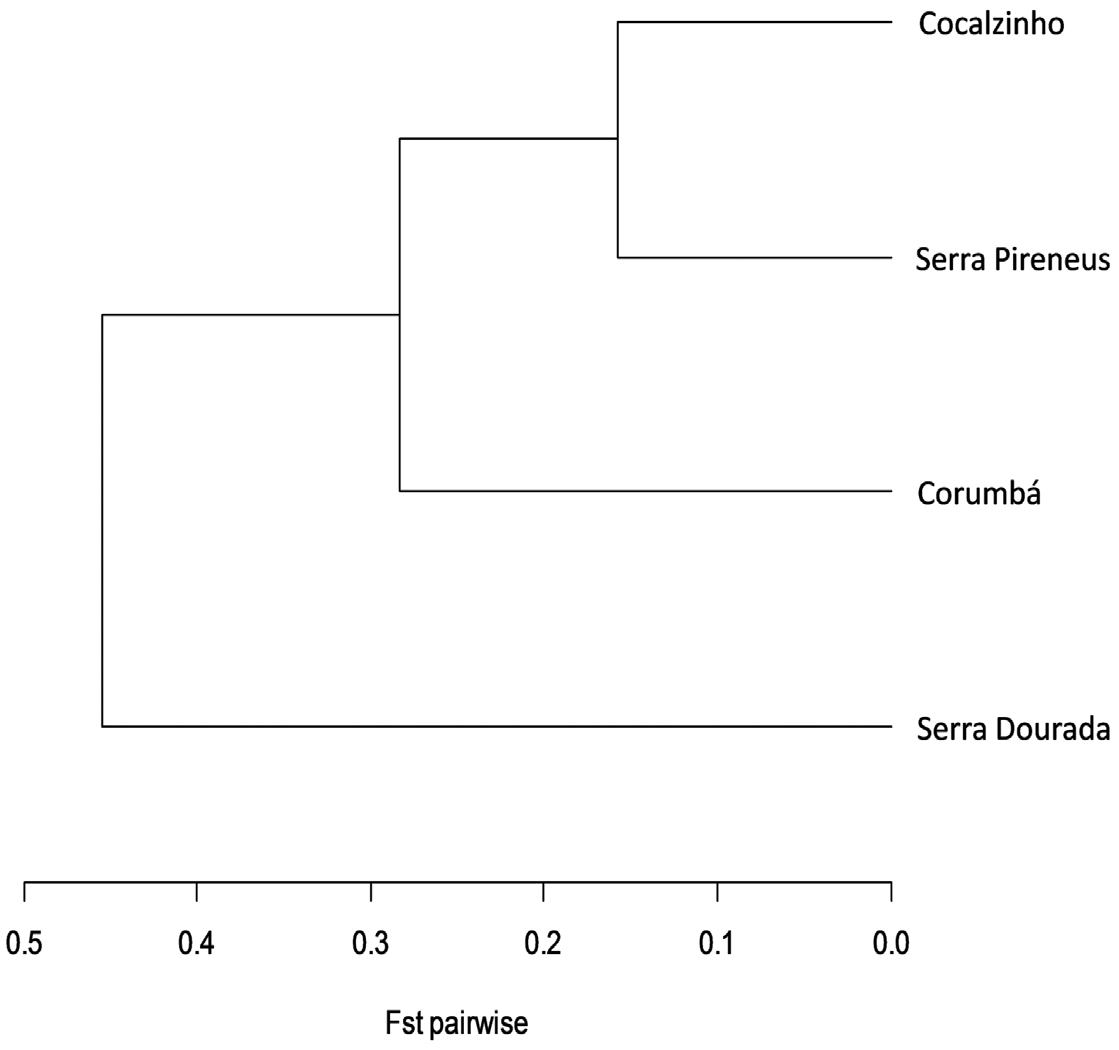
Dendrogram constructed from the F_ST_ pairwise matrix of four populations of *Manihot irwinii* sensu lato evaluated with seven microsatellite markers. The cophenetic correlation was 0.8933.

This pattern of genetic differentiation between populations can be observed in the dendrogram constructed from the F_ST_ pairwise matrix, which clearly points out the separation from the population collected in Serra Dourada in a distinct group (Figure 6). This result, associated with the strong genetic structure detected, suggests that populations of *Manihot irwinii* sensu lato are quite distinct from each other, particularly from *Manihot pulchrifolius* (population from Serra Dourada), which seems to be genetically isolated.

**Table 2.** Genetic diversity parameters estimated for four populations of *Manihot irwinii* sensu lato, based on seven microsatellite loci.

Municipality	A	AR	He	Ho
Mossâmedes (n = 29)	3.714	3.38	0.454	0.389
Corumbá de Goiás (n = 33)	2.429	2.17	0.321	0.368
Pirenopólis (n = 37)	4.286	3.76	0.578	0.574
Cocalzinho de Goiás (n = 27)	3.143	2.952	0.434	0.464

n: number of individuals selected in each population; A: average number of alleles per locus; AR: rarefied allelic richness; He: expected heterozygosity under Hardy-Weinberg equilibrium; Ho: observed heterozygosity.

## Supplementary Material

XML Treatment for Manihot pulchrifolius
